# Frequency of Hyponatremia in Patients With Chronic Liver Disease Presenting to the Outpatient Department: A Cross-Sectional Study

**DOI:** 10.7759/cureus.106467

**Published:** 2026-04-05

**Authors:** Nadeem Bajkani, Kamran Ali Memon, Syed Zahid Hussain, Salman Ahsam, Ghulam Abbas, Muhammad Jaffar Odhano, Raja Taha Yaseen Khan

**Affiliations:** 1 Gastroenterology and Liver Transplant, Gambat Institute of Medical Sciences, Gambat, PAK; 2 Gastroenterology, Gambat Institute of Medical Sciences, Gambat, PAK; 3 Gastroenterology, Social Security Hospital, Faisalabad, PAK; 4 Gastroenterology and Hepatology, Allama Iqbal Medical College, Lahore, PAK; 5 Internal Medicine, Ziauddin University, Sukkur, PAK; 6 Gastroenterology, Sindh Institute of Urology and Transplantation (SIUT), Karachi, PAK

**Keywords:** child–turcotte–pugh (ctp) score, chronic liver disease (cld), cirrhosis, hyponatremia, model for end-stage liver disease (meld) score

## Abstract

Background: Hyponatremia is a common and clinically significant complication in patients with chronic liver disease (CLD), often associated with increased morbidity and progression to hepatic decompensation. While its prevalence in hospitalized cirrhotic patients is well documented, data from outpatient populations remain limited.

Objective: Therefore, the aim of our study was to determine the frequency of hyponatremia in patients with CLD presenting to the outpatient department.

Methods: This cross-sectional study was conducted at the outpatient department of Medical Unit V, Jinnah Sindh Medical University, between January and December 2019. A total of 224 patients with clinically and/or radiologically diagnosed CLD were included using non-probability consecutive sampling. Patients with conditions or medications known to cause hyponatremia were excluded. Serum sodium levels were used to classify hyponatremia as mild (130-134 mmol/L), moderate (125-129 mmol/L), and severe (<125 mmol/L). Data on demographic, clinical, and laboratory parameters were collected and analyzed using IBM SPSS Statistics for Windows, Version 27 (Released 2019; IBM Corp., Armonk, New York, United States).

Results: Out of 224 patients, 94 (42%) were found to have hyponatremia, with 52 (23.2%) having mild hyponatremia, 29 (12.9%) moderate hyponatremia, and 13 (5.8%) severe hyponatremia. The most common cause of CLD was hepatitis C (34.8%), followed by hepatitis B (27.2%) and metabolic associated fatty liver disease (17.4%). Hyponatremia was significantly associated with lower hemoglobin and serum albumin levels, presence of ascites, and elevated INR, total bilirubin, Child-Turcotte-Pugh (CTP) score, and model for end-stage liver disease score (p<0.05 for all), indicating more advanced liver disease.

Conclusion: Hyponatremia is prevalent in stable CLD patients presenting to outpatient clinics and correlates strongly with indicators of disease severity. Routine monitoring of serum sodium levels in outpatient settings may enable early identification of high-risk individuals and timely therapeutic interventions to delay hepatic decompensation.

## Introduction

Chronic liver disease (CLD) encompasses a spectrum of progressive hepatic disorders characterized by long-term inflammation, fibrosis, and eventual cirrhosis [1]. Globally, it constitutes a significant health challenge, contributing to substantial morbidity and mortality. In low- and middle-income countries [2]. In Pakistan, the burden is amplified due to the high prevalence of chronic hepatitis B and C infections, alcohol-related liver injury, and the rising incidence of metabolic associated fatty liver disease (MAFLD) due to metabolic syndrome [3-6].

Among the complications of CLD, hyponatremia is a frequent and clinically significant electrolytic disturbance [7]. It has been defined as a serum sodium level less than 135 mmol/L and is especially prevalent in advanced disease in cirrhosis patients [8]. Hyponatremia in the context of CLD is often dilutional in origin and due to depressed renal clearance of free water rather than sodium loss per se. This pathophysiologic phenomenon is mediated primarily by portal hypertension, resulting in splanchnic vasodilation, reduced effective circulating volume, and thus compensatory activation of the renin-angiotensin-aldosterone system, sympathetic nervous system, and non-osmotic release of antidiuretic hormone (ADH). All of these hormonal effects promote sodium and water retention and dilutional hyponatremia [9].

Clinical features of hyponatremia in CLD comprise neuropsychiatric symptoms such as lethargy, confusional states, unsteadiness and increased vulnerability to hepatic encephalopathy. Moderate hyponatremia and severe hyponatremia have greater hospitalization risks, spontaneous bacterial peritonitis, hepatorenal syndrome, and mortality [7,9].

In South Asian nations such as Pakistan, where the disease burden of the liver remains significant and access to health care is often limited, it becomes important to identify and estimate the prevalence of hyponatremia in outpatient settings. Although hyponatremia is well-recognized in patients with decompensated CLD admitted to the intensive care units, its frequency in stable outpatients with CLD is under-reported. Identifying this electrolyte imbalance in the outpatient setting is crucial because even mild hyponatremia can predict poor outcomes. Prompt recognition in stable patients may aid in the management to delay decompensation, reduce morbidity and improve quality of life. This study fills a knowledge gap by assessing the burden of hyponatremia in a relatively stable CLD cohort. Therefore, the main aim of this study is to determine the frequency of hyponatremia in patients with CLD presenting to the outpatient department.

## Materials and methods

Study methodology

After the approval from the ethical review board (A-1071), this cross-sectional observational study was carried out at the outpatient department of gastroenterology, Isra University, Hyderabad from January 2019 to December 2019. All the patients aged ≥18 years with a diagnosis of CLD defined by the presence of three or more of the following findings such as altered echo texture of liver; irregular liver margins; spleen size more than 12 cm; portal vein diameter more than 12 mm and presence of free fluid in the abdomen on ultrasound were enrolled in the study [1]. Patients with acute liver failure (defined as the presence of hepatic encephalopathy and an INR ≥1.5 within 26 weeks of symptom onset in the patients with pre-existing CLD or cirrhosis) [10]; those with end-stage renal disease or chronic kidney disease with estimated glomerular filtration rate of less than 30ml/min; those on medications known to cause hyponatremia (e.g., diuretics, SSRIs, carbamazepine); patients with underlying congestive heart failure or active or untreated malignancy and those with a history of recent hospitalization (within past 30 days were excluded from the study.

Sample size estimation

Assuming an expected prevalence of 30 % of hyponatremia in patients with CLD with a 95% confidence level and 6% absolute precision and keeping a margin of error of 6%, a total of 224 patients were enrolled through the method of non-probability consecutive sampling [11].

Data collection procedure

After obtaining informed consent, all the patients with CLD were recruited. Demographic and clinical data were obtained from each patient such as age, gender, etiologies of CLD and the presence or absence of ascites or encephalopathy at presentation. Blood samples were collected to assess laboratory parameters such as hemoglobin, total leucocyte count, platelet count, liver function tests, renal function tests, serum electrolytes such as sodium and potassium, coagulation profile, Child-Turcotte-Pugh (CTP) score, model for end-stage liver disease (MELD) score and presence of ascites or encephalopathy. Hyponatremia was defined as mild (130-134 mmol/L), moderate (125-129 mmol/L) and severe (<125 mmol/L) hyponatremia.

Data analysis procedure

Data was entered and analyzed using IBM SPSS Statistics for Windows, Version 27 (Released 2019; IBM Corp., Armonk, New York, United States). Categorical variables were expressed as frequencies and percentages, while continuous variables were expressed as Mean ± SD. A chi-square test was applied to assess the association between hyponatremia and categorical variables like gender, CTP class and ascites. Independent student t-test was used for the comparison of continuous variables. A p-value <0.05 was considered statistically significant.

## Results

A total of 224 patients with CLD were enrolled in the study. Among them, 123 (55%) were male. The mean age of the studied population was 51.3 ± 10.2 years. The most common cause of CLD was hepatitis C (78 (34.8%)), followed by hepatitis B (61 (27.2%)), MAFLD (39 (17.4%)), alcoholic liver disease (31 (13.8%)), autoimmune hepatitis (9 (4.1%)) and Wilson disease (6 (2.7%)). Among the participants of this study, 94 (42%) patients were found to have hyponatremia. Among them, 52 (23.2%) patients had mild hyponatremia, 29 (12.9%) had moderate hyponatremia (125-129 mmol/L) while 13 (5.8%) patients were classified as having severe hyponatremia (serum sodium <125 mmol/L). Ascites was observed in 107 (47.8%) patients. Mean hemoglobin was 9.8 ± 1.6 g/dL, TLC was 6.9 ± 2.4 x 10^9^/L, platelets were 55 ± 23 x 10^9^/L, serum bilirubin was 2.1 ± 1.1 mg/dl, serum albumin was 2.6 ± 0.48 g/dl, INR of 1.46 ± 0.2, CTP score of 7± 2 and MELD score of 16.4 ± 3.1 (Table 1).

**Table 1:** Baseline characteristics of the studied population (n: 224) INR: International Normalized Ratio; CTP: Child-Turcotte-Pugh score; MELD: Model for End-Stage Liver Disease; MAFLD: Metabolic Associated Fatty Liver Disease

Variables		n (%)
Gender	Male	123 (55)
	Female	101 (45)
Cause of chronic liver disease	HCV	78 (34.8)
	HBV	61 (27.2)
	MAFLD	39 (17.4)
	ALD	31 (13.8)
	AIH	9 (4.1)
	Wilson disease	6 (2.7)
Hyponatremia	Present	94 (42)
	Absent	130 (58)
Severity of hyponatremia	Mild	52 (23.2)
	Moderate	29 (12.9)
	Severe	13 (5.8)
Ascites	Yes	107 (47.8)
	No	117 (52.2)
Variables		Mean + S.D.
Mean age (years)		51.3 ± 10.2
Hemoglobin (g/dL)		9.8 ± 1.6
Total leucocyte count (x10^9^/L)		6.9± 2.4
Platelet count (x10^9^/L)		55 ± 23
Serum creatinine (mg/dl)		0.96 ± 0.38
Total bilirubin (mg/dl)		2.1 ± 1.1
Alanine transaminase (ALT) (IU)		55.8 ± 23.9
Aspartate transaminase (AST) (IU)		47.3 ± 33.1
Albumin(g/dl)		2.8 ± 0.48
INR		1.46 ± 0.2
CTP score		7± 2
MELD score		16.4 ± 3.1

On comparative analysis, patients with hyponatremia had significantly lower mean hemoglobin (p=0.037) and serum albumin levels (p≤0.001) compared to those with normal sodium levels. Moreover, patients with hyponatremia were more likely to present with ascites (p≤0.001) and also had higher INR (p=0.002), total bilirubin (p=0.027), CTP (p≤0.001) and MELD scores (p≤0.001) indicating worse liver function (Table 2).

**Table 2:** Comparison of different variables in terms of presence or absence of hyponatremia (n: 224) INR: International Normalized Ratio; CTP: Child-Turcotte-Pugh score; MELD: Model for End-Stage Liver Disease

Variable		Hyponatremia		p-value
		Present (N=94) N (%)	Absent (N=130) N (%)	
Gender	Male	57 (60.6)	66 (50.7)	0.043
	Female	37 (39.4)	64 (49.3)	
Ascites	Yes	76 (80.9)	31 (23.8)	≤0.001
	No	18 (19.1)	99 (76.2)	
Age (years)		51.7 ± 9.4	50.4 ± 10.8	0.679
Mean hemoglobin (g/dl)		9.3 ± 1.3	10.7± 1.6	0.037
Total leucocyte count (x10^9^/L)		7.1 ± 1.3	6.63 ± 0.9	0.152
Platelet count (x10^9^/L)		73 ± 21	69 ± 27	0.699
Serum creatinine (mg/dl)		0.94 ± 0.12	0.87 ± 0.17	0.073
Total bilirubin (mg/dl)		2.3 ± 0.64	1.7 ± 0.71	0.027
Alanine transaminase (IU/L)		54 ± 26	57 ± 24	0.312
Aspartate transaminase (IU/L)		47.5 ± 28.1	47.1 ± 23.1	0.63
Serum albumin (g/dl)		2.5 ± 0.7	3.1 ± 0.4	≤0.001
INR		1.46 ± 0.37	1.23 ± 0.27	0.002
CTP score		8.4 ±1.6	7.1 ± 1.2	≤0.001
MELD score		17 ± 4.7	13.6 ± 2.3	≤0.001

## Discussion

In this study, hyponatremia was a frequent abnormality in the CLD population presenting to the outpatient clinics, noticed in 42% of the studied population. Among them, 23.2% patients had mild, 12.9% moderate, and 5.8% had severe hyponatremia. This prevalence highlights the importance of routine electrolyte monitoring even in stable CLD patients, as hyponatremia is often an early marker of disease progression and is associated with increased morbidity. Our findings are similar to the previous studies conducted worldwide, both on inpatient and outpatient populations. A study by Angeli et al. described a 22% prevalence of hyponatremia (serum sodium <130 mmol/L) in hospitalized cirrhotic patients, but noted even higher rates when using a cut-off of <135 mmol/L, up to 50% in some cohorts, especially in those with ascites and advanced liver dysfunction [12]. Although our cohort consisted of relatively stable outpatients, the high frequency (42%) supports the global evidence indicating that hyponatremia is not exclusive to decompensated or hospitalized individuals.

In an Indian prospective observational study, Reddy et al. observed a prevalence of hyponatremia of 53%, very closely agreeing with our result. They too demonstrated an important association between hyponatremia and higher CTP score, ascites, and hepatic encephalopathy, essentially the same associations that we demonstrated in our population [13]. This implies the common pathophysiological mechanism between populations based on portal hypertension, splanchnic vasodilation, and non-osmotic release of ADH [14].

In our study, we observed that hyponatremia is associated with higher MELD and CTP scores, thus predicting adverse outcomes in patients with CLD. A study done by Reddy et al. in an Indian population observed that dilutional hyponatremia markedly predicted adverse outcomes with higher hospitalization rates and mortality. They emphasized that mild levels of hyponatremia are capable of raising the chances of hepatic encephalopathy and compromised quality of life [15].

The Italian study by Bernardi et al. stressed that hyponatremia is frequently under-recognized in outpatient hepatology clinics, although it has long been known to be associated with hepatic decompensation [16]. This study emphasized the routine outpatient monitoring to detect serum sodium abnormalities, which is consistent with the suggestion of our study that sodium monitoring must be included in outpatient management algorithms, particularly in cases with ascites and hypoalbuminemia.

Notably, patients with hyponatremia in the current study also had significantly decreased serum albumin and hemoglobin levels. This finding may indicate an end-stage disease with systemic inflammation, malnutrition, and portal hypertension with fluid retention. Comparable correlations have been observed in the study conducted by Kim et al. in Korea, in which decreased albumin and increased INR were predictors of hyponatremia among cirrhotic patients [17].

Yet another worldwide multicenter study, the CANONIC study, similarly stressed the fact that hyponatremia among cirrhotics is associated with circulatory dysfunction and has an impact on acute-on-chronic liver failure (ACLF) risk stratification [18]. Though the present study did not measure ACLF directly, the results supported the idea that hyponatremia is perhaps a sentinel marker with the potential to detect, earliest, systemic complications and the process of decompensation.

This study had certain limitations. First, the cross-sectional study design does not allow for causal inference or longitudinal follow-up on the progression of disease in patients with hyponatremia. Second, the study was conducted at a single center, which might prevent generalizability of the results to the whole population. One of the specific strengths of this work is an exploration of the outpatient population, otherwise often understudied with respect to disturbances of electrolytes. Most available published data have been based on hospital or critically ill patients. These results provide valuable information regarding the recognition of hyponatremia at an early level, with the possibility of intervention at the correct time and prevention of hepatic decompensation.

## Conclusions

This study predicted a high frequency of hyponatremia among patients with CLD presenting to the outpatient department with a significant association of hyponatremia with more advanced liver disease as indicated by elevated CTP and MELD scores. These findings emphasize the importance of routine monitoring of serum sodium levels, even in apparently stable CLD outpatients, to enable early detection of hepatic decompensation and timely intervention. Given the strong association of hyponatremia with poor clinical parameters and disease severity, its presence should be considered a sentinel marker for prognostication and therapeutic planning in chronic liver disease management. Multicentered longitudinal studies should be conducted to assess the influence of improving hyponatremia on clinical outcomes and survival in this population.
